# Icariin Prevents Cartilage and Bone Degradation in Experimental Models of Arthritis

**DOI:** 10.1155/2016/9529630

**Published:** 2016-04-20

**Authors:** Chen Chao Wei, Dai Qi Ping, Fan Tian You, Chen Yong Qiang, Che Tao

**Affiliations:** Department of Orthopaedics and Traumatology, Shanghai Municipal Hospital of Traditional Chinese Medicine, Shanghai University of TCM, Shanghai 200071, China

## Abstract

*Background*. Icariin (ICA) is an active compound extracted from* Epimedium brevicornum* Maxim. Previous reports have shown that icariin has a clinically significant therapeutic effect on rheumatoid arthritis. However, little is known about the mechanism by which icariin inhibits cartilage and bone degradation.* Methods*. New Zealand rabbits were immunized with antigen-induced arthritis (AIA) and treated with icariin. Joint tissues from rabbits were studied by histological analysis, transmission electron microscopy (TEM), and micro-CT. The expression levels of receptor activator of nuclear factor-B ligand (RANKL) and osteoprotegerin (OPG) in joint tissues were determined using immunohistochemistry and real-time PCR analysis.* Results*. Histological analysis and TEM sections of cartilage in the ICA treated group showed a low level of chondrocyte destruction. Micro-CT analysis showed that the bone mineral density value and bone structural level in ICA treated rabbits were significantly higher compared with those in the AIA group. Immunohistochemistry and real-time PCR analysis showed that icariin treatment reduced RANKL expression and enhanced OPG expression levels, as compared to the AIA group.* Conclusion*. These data indicate that ICA suppresses articular bone loss and prevents joint destruction. This study also determined that ICA regulated articular bone loss in part by regulating RANKL and OPG expression.

## 1. Introduction

Rheumatoid arthritis (RA) is characterized by a chronic inflammatory joint disease which eventually leads to juxta-articular destruction and functional disability [1]. The destruction of bone and cartilage has previously been reported to result from a large proportion of T-cells and B-cells invading the synovial tissue [2]. In addition, bone loss in RA has been linked to an imbalance between bone formation and resorption [3]. Chronic synovitis may contribute to the production of inflammatory factors such as IL-1*β*, IL-6, IL-17, and TNF-*α*. These inflammatory factors in turn upregulate the expression levels of receptor activator of nuclear factor-B ligand (RANKL) and enhance bone resorption with a corresponding large increase in bone formation [4].

Recent studies have shown that the RANKL/osteoprotegerin (OPG) balance plays an important role in the cartilage and bone degradation seen in the development of RA. Any imbalance between RANKL and OPG may lead to osteoarticular pathology [5]. In RA, overexpression of RANKL can induce synovial macrophage differentiation into active osteoclasts, eventually leading to bone destruction. OPG prevents osteoclast differentiation and activation thereby neutralizing the receptor activation of RANKL [6]. An anti-RANKL antibody, which is currently being evaluated in clinical trials, has demonstrated efficacy for the treatment of osteoporosis in postmenopausal women. Treatment with an anti-RANKL antibody provided an improvement of bone mineral density (BMD) [7]. In summary, regulation of the RANKL/OPG balance not only prevents osteoporosis but also prevents joint destruction in RA patients.

Icariin (ICA) is an active compound extracted from the traditional Chinese herb* Epimedium brevicornum *Maxim. Icariin has been reported to have a variety of pharmacological actions, including effects on myocardial cells and cerebral cells. Extensive studies have shown that icariin exhibits anti-inflammatory and estrogen-like effects and immunoregulatory activities [8]. A previous report observed that icariin has a clinically significant and therapeutic effect on RA [9]. Chi et al. found that icariin prevents rheumatoid arthritis, which might be mediated by a decrease in the number of Th17 cells. In addition, icariin administration inhibited the production of IL-17 [10]. A recent study found that icariin stimulated bone formation and resorption by increasing proliferation and the differentiation of normal human osteoblast cells* in vitro* [11]. Sun et al. showed that the ability of icariin to inhibit the protease activity of cathepsin K is responsible in part for its ability to prevent cartilage and bone degradation [12]. Wang et al. found that icariin has a reparative effect on rapid palatal expansion induced root resorption in rats, by regulating the OPG/RANKL expression ratio and regulating osteoclast differentiation [13].

Antigen-induced arthritis (AIA) is a well-established animal model of chronic arthritis. X-ray radiography and micro-CT analysis showed that the inflammatory arthritis that develops in AIA is accompanied by severe articular bone loss [14]. In contrast to other arthritis models of RA, AIA is induced by T cell-cognate immunity in response to a known exogenous antigen. The mechanisms responsible for T cell mediated pathology during adjuvant arthritis are clear [15]. Thus, our experimental model offers a valuable opportunity to enhance our understanding of the underlying mechanisms by which icariin prevents cartilage and bone degradation. We hypothesized that icariin might mediate its effects by regulating the RANKL/OPG balance and alleviating rheumatoid arthritis. In the present study, we investigated the effect of the icariin on cartilage and bone destruction during AIA. Our results provide further experimental evidence for the clinical use of icariin in the treatment of RA.

## 2. Methods

### 2.1. Drugs and Animals

Icariin was purchased from the Jimian Plant Extraction Technology Co. Ltd., Shanghai, China. Indomethacin was purchased from Shanghai Xinyi Pharmaceutical Co. Ltd., China. 25 mg tablets of indomethacin were administered as a positive control. New Zealand rabbits with a body weight of 2–2.5 kg were obtained from the Shanghai Laboratory Animal Center of the Chinese Academy of Sciences.

AIA rabbits were induced as in previous studies [16]. Briefly, AIA rabbits were induced by immunizing them with 4 mg ovalbumin (OVA) in Freund's complete adjuvant (Sigma Chemical Co., St. Louis, MO, USA). This injection procedure was repeated once a week for 2 weeks, followed 4 weeks later by boosts of 1 mg of OVA once a week. Six weeks after the last booster injection, AIA rabbits were graded by scoring clinical signs according to criteria described in earlier studies [17]. The rabbits that had clinical scores higher than six were deemed as having arthritis and were then randomly divided into three groups: the AIA group (*n* = 6), the ICA group (*n* = 6), and the control group (*n* = 6). The normal group of rabbits (*n* = 6) did not undergo a procedure.

The rabbits in the ICA group received intragastric (IG) administrations of ICA at 60 mg/kg per day. Rabbits in the control group received IG administrations of indomethacin at 3 mg/kg per day. Both dosages are equivalent to 10 times the clinical dosage for a 60 kg adult. The drug treatment was performed over a period of 3 months. Rabbits in the normal and AIA groups received an equal volume of water. Rabbits were then sacrificed after 3 months. Articular cartilage and knee joints were collected from rabbits after being euthanized. All procedures using animals received prior approval by the committee for Animal Experiments of the University Medical Center, Shanghai, China.

### 2.2. Histological Analysis

For histological analysis of bone, the left proximal tibia was dissected and fixed in 4% paraformaldehyde and then was decalcified in 10% EDTA for about 3 weeks. The left proximal tibia was then dehydrated in a graded series of ethanol solutions and then cleared in amyl acetate. Tibias were then cut into 5 *μ*m thick sections for histomorphometry. The sections were stained with hematoxylin and eosin (H&E) for morphological measurements. H&E stained slides were subsequently stained with Safranin-Orange to assess glycosaminoglycan content. These histological sections were evaluated using a modified Mankin grading system [18] by three blinded, independent pathologists.

### 2.3. Transmission Electron Microscopy

After demineralization, cartilage tissue specimens were fixed in 2.5% glutaraldehyde in Sorenson's phosphate buffer, rinsed in buffer, and then decalcified in a controlled manner using 10% EDTA in order to remove organic material. Specimens were dehydrated using a series of ethanol solutions (70%–100%). Each specimen was coated with Epon812 for use in transmission electron microscopy (TEM). Tissue sections were cut to a 60 nm thickness and then double stained sequentially using uranyl and phosphotungstic acid. The samples were then prepared for TEM in order to observe chondrocytes (HITACHI H-500, Japan). The numbers of mitochondria, Golgi bodies, and vacuoles were counted in at least 100 chondrocytes from each group [19].

### 2.4. Skeletal Phenotyping

The right distal femurs from rabbits were scanned using micro-CT (GE Locus SP) to assess bone mass, density, trabecular geometry, and microarchitecture. Bone mineral density (BMD), trabecular thickness (Tb.Th), bone volume (BV/TV), trabecular number (Tb.N), and trabecular separation (Tb.Sp) were computed for all specimens.

### 2.5. Immunohistochemical Analysis of RANKL and OPG Expression in Proximal Tibia

Tibias were cut into 5 *μ*m thick sections for immunohistochemical examinations. To reduce endogenous peroxide activity, sections were quenched with 3% hydrogen peroxide for 10 min and then blocked in Tris buffered saline containing 3% normal goat serum. The sections were incubated with primary mouse anti-rabbit antibodies for OPG (1 : 50 dilution) and RANKL (1 : 100 dilution) (Santa Cruz, CA) at 4°C for 24 h, followed by biotin-labeled secondary antibodies. After staining the nucleus with 4′,6-diamidino-2-phenylindole, the slides were then stained with Cy3-labeled secondary antibodies for 1 h at 37°C. The slides that were stained with PBS instead of the primary antibody were used as negative controls.

### 2.6. RT-PCR Analysis of RANKL mRNA and OPG mRNA

Trizol reagent was used to extract total RNA from proximal tibias and cartilage. 1 *μ*g of total RNA was reverse transcribed using the Revert Aid First Strand cDNA synthesis kit (Bio-Rad) [20] and subsequently amplified by real-time PCR in a Rotor-Gene 2000 system (Australia) [21]. Relative quantitation was calculated using delta cycle threshold relative quantitation. The primers were designed as follows:

*RANKL*

 Forward primer 5′-TTTGCAGGACTCGACTCTGGAG,
 Reverse primer 5′-TCCCTCCTTTCATCAGGTTATGAG;

*OPG*

 Forward primer 5′-ATCATTGAATGGACAACCCAGG,
 Reverse primer 5′-TGCGTGGCTTCTCTGTTTCC;

*GAPDH*

 Forward primer 5′-GATCGTGGAAGGGCTAATGA,
 Reverse primer 5′-GACTTTGCCTACAGCCTTGG.

### 2.7. Statistical Analysis

All values were expressed as the mean and standard deviation (SD). Measurement data were analyzed using a one-way analysis of variance (ANOVA, SPSS 11.0). Rank data were treated using ridit analysis. A result of *p* < 0.05 was considered to be statistically significant.

## 3. Results

### 3.1. Icariin Reduces Arthritis Cartilage Degradation

**Figure 1:**
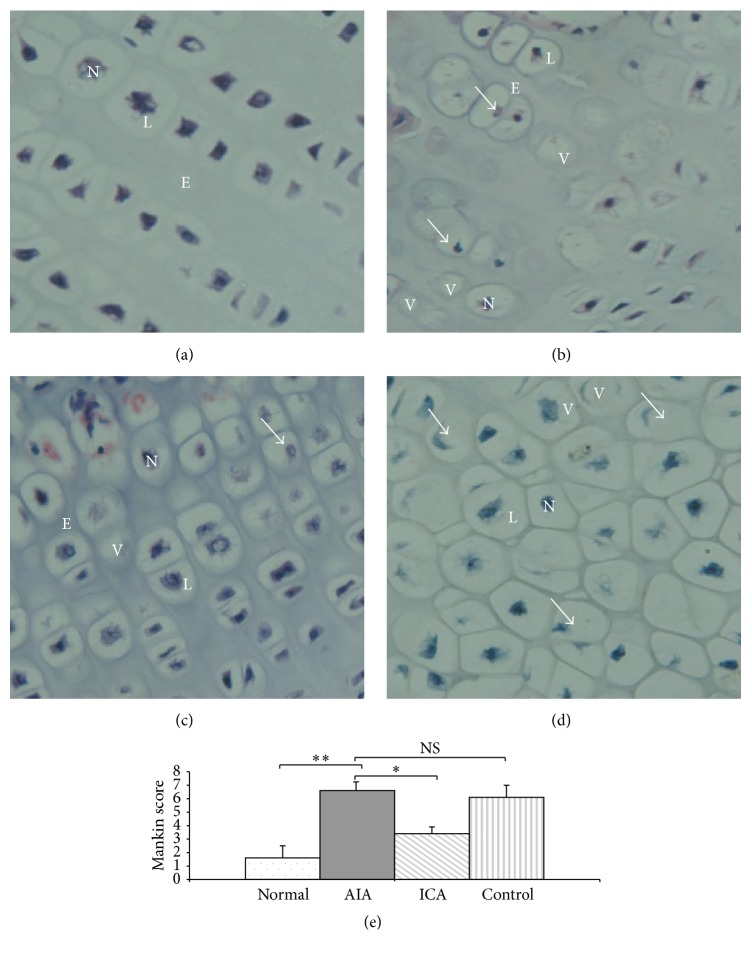
Histological evaluation of rabbit knee joint cartilage stained with haematoxylin and eosin. (a) Normal group showing the chondrocytes were arranged in a straight line and located centrally within its lacunae. These chondrocytes showed centrally located nuclei, fine granular cytoplasm that contains few discrete vacuoles, and were surrounded by extracellular matrix. (b) AIA group: the chondrocytes (arrows) showed significant destruction along with pyknotic nuclei, no clear lacunae, and many vacuoles inside the lacunae. (c) ICA group showing destroyed chondrocytes (arrows). Nuclei were not centrally located and within a vacuole inside the lacunae. (d) Control group: the disrupted chondrocytes showed that many atrophic nuclei were not centrally located and some had no nuclei (arrows) (original magnification ×300). L, lacunae; N, nuclei; E, extracellular matrix; V, vacuoles. (e) The Mankin grading system was used for the evaluation of articular cartilage degeneration. In the AIA group, higher Mankin score values were obtained with increasing cartilage degeneration (*p* < 0.01). However, there was a lower mean score in the ICA group compared with that in the AIA group (*p* < 0.05). Results are presented as the mean ± SD. *n* = 6  ^*∗*^ *p* < 0.05, ^*∗∗*^ *p* < 0.01 versus corresponding group.

Examination of cartilage from the normal group demonstrated that the chondrocytes were arranged in a group and were centrally located within its lacunae. These chondrocytes showed centrally located nuclei, finely granular cytoplasm that contains discrete vacuoles, and were surrounded by extracellular matrix (Figure 1(a)). In contrast, the cartilage from AIA group rabbits showed highly destroyed chondrocytes and pyknotic nuclei with no clear lacunae. In addition the cells displayed a shrunken cytoplasm with many vacuoles and were surrounded by a small amount of cartilage matrix (Figure 1(b)). However, chondrocytes were almost completely preserved in the ICA treated groups. We have observed that enlarged chondrocytes were arranged in groups and situated centrally within their lacunae. The cells were surrounded by faint layer of extracellular matrix. ICA chondrocytes contained fine granular cytoplasm that contains discrete vacuoles (Figure 1(c)). Furthermore, the IND positive control group showed that many disrupted chondrocytes were present within its disrupted lacunae (Figure 1(d)).

The Mankin grading system is used for evaluation of articular cartilage degeneration. This system uses the parameters of cartilage surface structure, chondrocyte loss, glycosaminoglycan reduction, and tidemark integrity as the basis for establishing a score. In the AIA group, higher Mankin score values were obtained with increasing cartilage degeneration. There were significant differences between the normal group and the AIA group (*p* < 0.01). However, there was a lower mean score in the ICA group compared with that in the AIA group (*p* < 0.05). There were no statistically significant differences in the Mankin score between the IND group and the AIA group (*p* > 0.05) (Figure 1(e)).

### 3.2. Icariin Protects Chondrocytes from Damage

**Figure 2:**
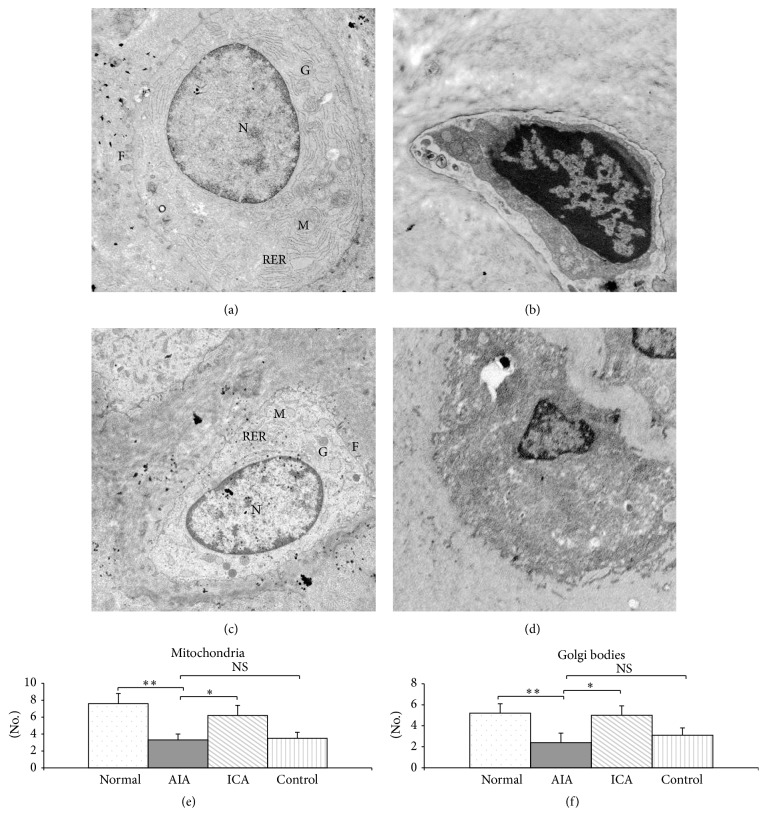
TEM micrographs of chondrocytes and numbers of organelles in chondrocytes were counted to evaluate chondrocytes degeneration. (a) Normal group, (b) AIA group, (c) ICA group, and (d) control group (×10,000 magnification). F, filopodia; G, Golgi; N, euchromatin nucleus; M, mitochondria; RER, rough endoplasmic reticulum. The numbers of mitochondria (e) and Golgi bodies (f) were significantly decreased in the AIA group compared with those in the normal group (*p* < 0.01), whereas the numbers of mitochondria and Golgi were greatly increased in the ICA group (*p* < 0.05). However, when compared with the AIA group, the levels present in the IND positive control rabbits did not attain statistical significance. Results are presented as mean ± SD. *n* = 6  ^*∗*^ *p* < 0.05, ^*∗∗*^ *p* < 0.01 versus corresponding group.

Representative TEM micrographs of chondrocytes are shown in Figure 2. TEM demonstrated that a normal cytoplasm contains a euchromatic cell nucleus. A region of their plasma membranes bears some relatively long filopodia. In addition, the chondrocyte cytoplasm contained well-developed rough endoplasmic reticulum, numerous well shaped Golgi apparatus, and mitochondria (Figure 2(a)). Chondrocytes in the AIA group displayed a different ultrastructure. AIA chondrocytes had irregular outlines. Typical findings in AIA chondrocytes were a plasma membrane which bears some relatively short filopodia and abundant vacuoles in the cytoplasm, whereas rough endoplasmic reticulum, Golgi apparatus, and mitochondria were strongly reduced (Figure 2(b)). However, TEM micrographs of deep zone chondrocytes in the ICA treated group showed that their cytoplasm contains a euchromatic nucleus. A strong reduction of the number of vacuoles, an abundant presence of rough endoplasmic reticulum, well defined Golgi bodies, and mitochondria were observed in ICA chondrocytes (Figure 2(c)). In addition, the filopodia present on the surface of the chondrocytes were larger and wider in the ICA group compared with that in the AIA group. Chondrocytes in the IND positive control rabbits showed irregular hyperchromatic nuclei surrounded by increased number of vacuoles. In contrast, the number of organelles present in the IND chondrocytes was strongly reduced.

The numbers of mitochondria and Golgi bodies were counted to evaluate chondrocytes degeneration. Both the numbers of mitochondria and Golgi bodies were significantly decreased in the AIA group compared with those in the normal group (*p* < 0.01), whereas the numbers of mitochondria and Golgi were greatly increased in the ICA group (*p* < 0.05). However, while the number of mitochondria and Golgi in IND positive control rabbits increased, the levels did not attain statistical significance when compared with the AIA rabbits group (Figures 2(e) and 2(f)).

### 3.3. Effect of Icariin Inhibition in Knee Joint Bone Loss

**Figure 3:**
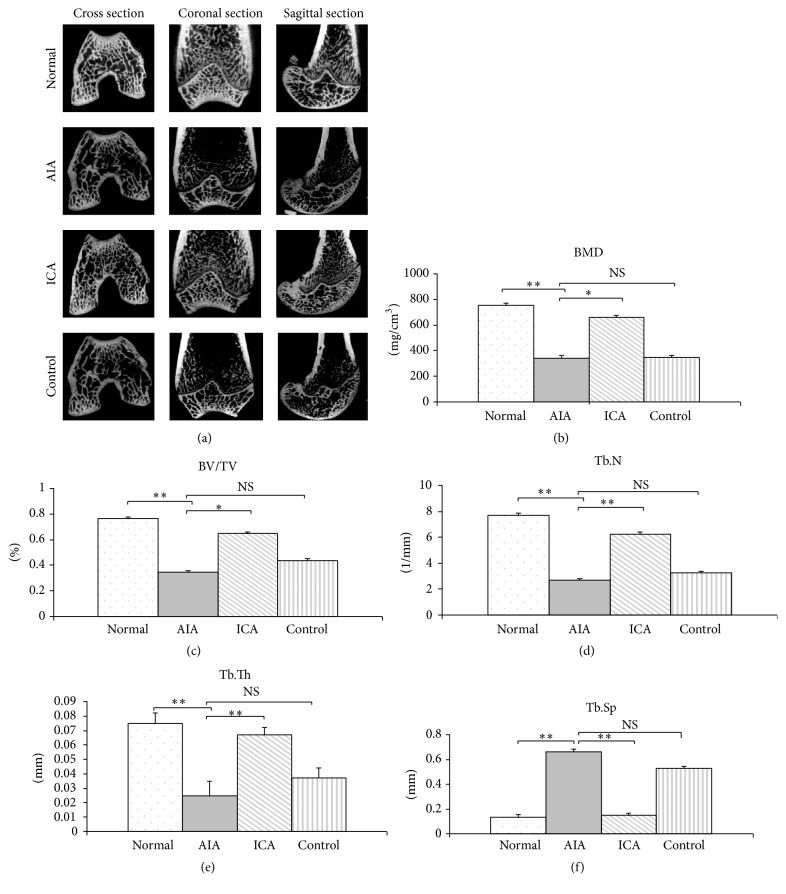
Effects of icariin on BMD and bone structural quality of rabbits measured by micro-CT. Representative *μ*-CT images of the distal femur (a). The BMD was significantly decreased in AIA rabbits as compared to the normal group rabbits. However, the BMD value in ICA treated rabbits was significantly higher compared with that in the AIA group and no change of BMD was found between the control group and the AIA group (b). There was a significant decrease in BV/TV (c), Tb.N (d), and Tb.Th (e) in the AIA rabbits as compared to the normal group. In addition there was a significant increase in Tb.Sp in the AIA group (f). Compared with AIA rabbits, the trabecular BV/TV (c), Tb.N (d), and Tb.Th (e) were significantly increased whereas the Tb.Sp (f) was significantly decreased in ICA treated rabbits. Results are presented as the mean ± SD. ^*∗*^ *p* < 0.05, ^*∗∗*^ *p* < 0.01 versus corresponding group.

The AIA group exhibited juxta-articular bone osteopenia as evidenced by micro-CT analysis (Figure 3(a)). The micro-CT images showed that lower bone volume in the trabecular bone of the distal femur and greater spacing between the trabecular bones were seen in the AIA group. Also, there was destruction of the three-dimensional trabecular bone structure in the distal femur. In addition, lower bone volume in the trabecular bones was seen in the IND positive control group. However, trabecular bone loss of the distal femur was inhibited in the ICA group.

The bone mineral density (BMD) was significantly decreased in AIA rabbits as compared to normal group rabbits (*p* < 0.01). However, the BMD value in ICA treated rabbits was significantly higher compared with that in the AIA group (*p* < 0.05). Further analysis of bone structural properties revealed that the trabecular bone microarchitecture in AIA rabbits as compared to the normal group rabbits was significantly decreased in BV/TV, Tb.N, and Tb.Th (*p* < 0.01) and had a significant increase in Tb.Sp (*p* < 0.01). Compared with AIA rabbits, ICA rabbits had a significant increase in trabecular BV/TV, Tb.N, and Tb.Th (*p* < 0.05). However, Tb.Sp was significantly decreased (*p* < 0.01) in ICA treated rabbits. Furthermore, we also observed that, compared with the AIA rabbits group, the BMD value in the IND positive control group did not attain statistical significance (Figure 3).

### 3.4. Decreased RANKL and Enhanced OPG Expression Are Associated with Icariin Therapy

**Figure 4:**
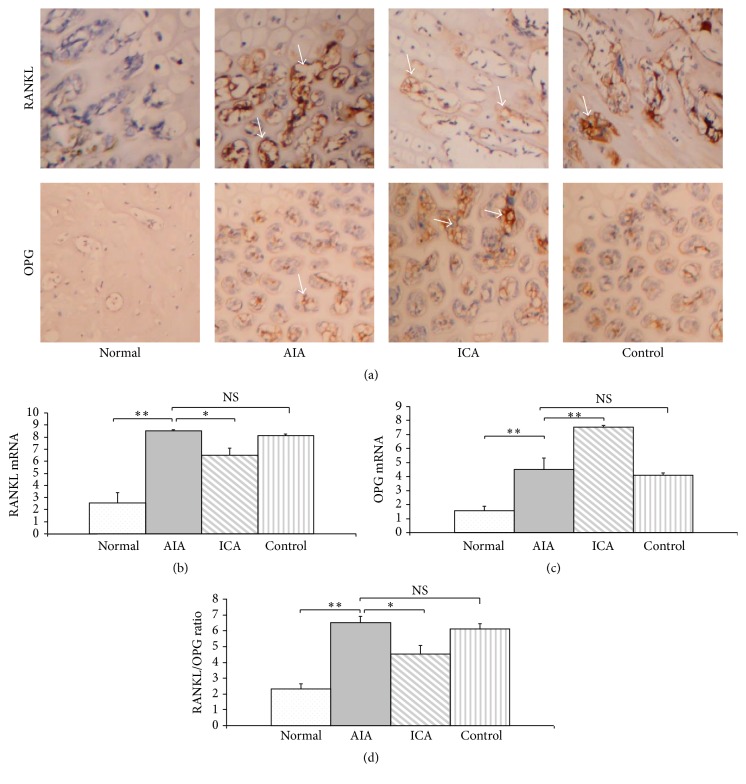
Decreased RANKL and enhanced OPG expression in articular cartilage is associated with icariin therapy. Icariin treatment reduced RANKL and enhanced OPG protein and mRNA expression in subchondral bone as shown by immunohistochemistry (a) and real-time PCR analysis (b, c). RANKL/OPG ratio (d). Results are presented as the mean ± SD. ^*∗*^ *p* < 0.05, ^*∗∗*^ *p* < 0.01 versus corresponding group. Arrow heads indicate positive staining in subchondral bone. Original magnification, ×400.

Immunohistochemical analysis of proximal tibia sections indicated that RANKL and OPG expressions were expressed at relatively low levels in normal group cartilage. However, AIA group rabbits exhibited higher expression of RANKL, with markedly higher intensity than that in normal animals. This increase in proximal tibia expression of RANKL was accompanied by an increase in intracellular RANKL expression in both cartilage and subchondral bone. There were very few cells expressing OPG in the cartilage of the AIA group. ICA treatment significantly reduced RANKL expression in the cartilage of the ICA group as compared with that in the AIA group. Nevertheless, OPG expression in the ICA group had higher positive staining compared with that seen in the AIA group. In the cartilage of the IND positive control rabbits, an increase in the intensity of the RANKL staining was observed as assessed by immunohistochemistry (Figure 4(a)).

The expressions of RANKL and OPG mRNA were analyzed by quantitative RT-PCR. The results showed that the expression of RANKL and OPG mRNA species was low in the normal group. However, the expression of RANKL increased significantly in the AIA group in comparison with that of normal group (*p* < 0.01). The expression of RANKL mRNA in the icariin treated rabbits was significantly decreased (Figure 4(b)). In contrast, an enhanced expression of OPG mRNA was expressed in the ICA group as compared with that in AIA group (*p* < 0.01) (Figure 4(c)). Although the RANKL mRNA levels in the IND positive control group showed a decreased expression as compared with that in the AIA group, there was no significant difference (*p* = 0.057). Moreover, when compared with the AIA group, the RANKL/OPG ratio showed a decrease in the ICA group (*p* < 0.05) (Figure 4(d)).

## 4. Discussion

Rheumatoid arthritis is a chronic systemic autoimmune inflammatory disease involving the breakdown of cartilage and adjacent bone tissue. Patients with RA develop bone destruction and poor joint function [22]. Thus, preventing cartilage and bone destruction is important for RA therapy [23]. In this study, we examined whether icariin has an inhibitory effect on cartilage and bone destruction in the AIA rabbit model of arthritis.

Articular cartilage is composed of chondrocytes surrounded by a dense extracellular matrix [24]. The damage to cartilage chondrocytes plays a crucial role in the pathogenesis of articular destruction in RA [25]. In our study, sections from rabbit cartilage of the normal group demonstrated that the chondrocytes were arranged in groups and were located centrally within its lacunae. In addition chondrocytes showed centrally located nuclei. Staining of chondrocytes revealed that they had a fine granular appearance that contained little vacuoles and were surrounded by extracellular matrix [26]. As reported previously, TEM micrographs revealed that the extracellular matrix in articular cartilage showed a fibrillary flatwork that was composed of denser and thicker fibrils. Type II collagen fibrils are the principal molecular component in ECM [27]. During cartilage degradation, chondrocytes manifest features typical of apoptosis, such as chondrocytes loss, a typically round or oval profile, membrane loss or blebbing, nuclear changes with aggregation of chromatin into dense areas, and peripheral segregation [28]. In our study, the TEM micrographs of arthritis cartilage showed chondrocyte degeneration in the AIA group and failure to develop significant numbers of important cytoplasmic organelles. The degenerating chondrocytes showed a strong reduction in cytoplasmic rough endoplasmic reticulum, Golgi apparatus, and mitochondria as reported previously [29]. However, the organelles in chondrocytes from the ICA treated groups were almost completely preserved. We have observed that enlarged chondrocytes with fine granular cytoplasm were arranged in groups and were localized centrally within its lacunae. TEM showed that ICA treated chondrocyte cytoplasm contains a centrally located euchromatic nucleus, an abundant presence of rough endoplasmic reticulum, well defined Golgi bodies, and mitochondria. Previous studies showed that icariin is a strong chondrocyte anabolic agent. Icariin affects chondrocyte proliferation and has anti-inflammatory effects. Liu et al. found that icariin can protect chondrocytes from lipopolysaccharide-induced inflammation and ECM degradation through inhibition of nitric oxide and matrix metalloproteinases [30]. Li et al. showed that icariin can enhance the integration of new-formed cartilage by increasing the synthesis of glycosaminoglycan and collagen type I [31]. The aforementioned results confirmed that ICA functions as a potential protector of cartilage tissue. These findings are of great interest considering that chondrocytes degeneration increased the risk of articular destruction in RA [32]. Inhibition of chondrocyte degeneration might be one of the important mechanisms by which ICA prevents articular destruction.

As reported previously by numerous studies, juxta-articular bone loss is a major unsolved problem in RA [33]. Micro-CT was used in order to investigate the effects of ICA on bone quality. The BMD value in ICA treated rabbits was significantly higher compared with that in the AIA group. Further analysis of bone structural properties indicated that ICA treated rabbits had significantly increased trabecular BV/TV, trabecular numbers, and thickness as compared to AIA rabbits. However trabecular spacing was significantly decreased in the ICA treated rabbits. These experimental results suggest that icariin may have therapeutic activity in knee joint bone loss. A recent study showed that icariin can suppress bone loss in OPG knockout mice through the activation of the *β*-catenin pathway and stimulation of osteoblast differentiation [34].

The integrity of the bone is maintained by the balance between osteoclastic bone resorption and osteoblastic bone formation [35]. Juxta-articular osteoporosis can occur as a result of increased local production of osteoclast activating cytokines [36], such as macrophage-colony stimulating factor and RANKL expressed by synovial fibroblasts. Both RANKL and M-CSF are crucial for differentiation and activation of osteoclasts [37]. Previous studies have showed that the RANKL and OPG expression levels represent a direct link between osteoblast maturation and bone erosion in RA [38]. Relatively high levels of soluble RANKL and decreased levels of OPG were detected in the synovial fluid of patients with rheumatoid arthritis [39]. Thus, the RANKL/OPG ratio is regarded as a good biomarker for the pathogenesis of RA bone destruction [40]. We evaluated the expression of RANKL and OPG in sections of proximal tibia to further understand the mechanisms underlying the protective effects of ICA against the bone loss. The experimental results show that the expression of RANKL was higher in the articular cartilage of rabbits in the AIA group. After administration of ICA we found lower levels of expression of RANKL and higher levels of expression of OPG compared to those seen in the AIA group. These results suggest that reduced expression of RANKL along with increased expression of OPG could lead to negative regulation of osteoclastogenesis and osteoclast activity [41]. In turn, this negative regulation would result in an inhibition of osteoclastic bone resorbing activity and eventually higher bone density in ICA treated rabbits. The results show that the primary mechanism by which icariin regulates juxta-articular bone density is through the regulation of the RANKL/OPG ratio by the inhibition of RANKL expression and stimulation of OPG production.

## 5. Conclusions

Taken together, the study results revealed for the first time that ICA could reduce arthritis cartilage degradation and prevent joint destruction. This study also determined that ICA inhibited juxta-articular bone loss in part by regulating the RANKL/OPG ratio.

## Abbreviations

ICA: Icariin
AIA: Antigen-induced arthritis
BMD: Bone mineral density
RANKL: Receptor activator of nuclear factor-B ligand
OPG: Osteoprotegerin
TEM: Transmission electron microscopy
IND: Indomethacin
IL-17: Interleukin-17
Th17: T-helper subset.
